# d-Borneol enhances cisplatin sensitivity via p21/p27-mediated S-phase arrest and cell apoptosis in non-small cell lung cancer cells and a murine xenograft model

**DOI:** 10.1186/s11658-022-00362-4

**Published:** 2022-07-26

**Authors:** Jinxiu Li, Jianmei Yuan, Yong Li, Jian Wang, Daoyin Gong, Qian Xie, Rong Ma, Jiajun Wang, Mihong Ren, Danni Lu, Zhuo Xu

**Affiliations:** 1 State Key Laboratory of Southwestern Chinese Medicine Resources, College of Pharmacy, Chengdu University of Traditional Chinese Medicine, Chengdu, China; 2 Department of Pathology, Hospital of Chengdu University of Traditional Chinese Medicine, Chengdu, China

**Keywords:** d-Borneol, Non-small cell lung cancer, Drug resistance, Cisplatin, Chemosensitizer, Cell cycle, Apoptosis

## Abstract

**Background:**

Cisplatin (CDDP) is commonly used to treat non-small cell lung cancer (NSCLC), but the appearance of drug resistance greatly hinders its efficacy. Borneol may promote drug absorption; however, synergism between borneol and CDDP in suppressing NSCLC is not clearly understood. Hence, we investigated borneol as a novel chemosensitizer to support chemotherapeutic efficacy and reduce side effects.

**Methods:**

We compared viability after exposure to d-borneol, l-borneol, and synthetic borneol in two NSCLC cell lines, A549 and H460, and selected the most sensitive cells. We then assessed synergy between borneol forms and CDDP in cisplatin-resistant NSCLC cells, H460/CDDP. Next, we identified effective concentrations and exposure times. Subsequently, we evaluated cell migration via wound healing and cell proliferation via clone formation assay. Then, we focused on P-glycoprotein (P-gp) function, cell cycle, apoptosis, and RNA sequencing to elucidate underlying molecular mechanisms for synergy. Finally, we used an H460/CDDP xenograft tumor model to verify antitumor activity and safety in vivo. Data were examined using one-way analysis of variance (ANOVA) for multiple datasets or *t*-test for comparisons between two variables.

**Results:**

d-Borneol was more effective in H460 than A549 cells. d-Borneol combined with CDDP showed greater inhibition of cell proliferation, migration, and clone formation in H460/CDDP cells than CDDP alone. RNA sequencing (RNA-seq) analysis identified differentially expressed genes enriched in cell cycle pathways. The impact of d-borneol on CDDP chemosensitivity involved arrest of the cell cycle at S phase via p27/p21-mediated cyclinA2/D3-CDK2/6 signaling and activation of intrinsic apoptosis via p21-mediated Bax/Bcl-2/caspase3 signaling. Further, d-borneol ameliorated drug resistance by suppressing levels and activity of P-gp. Cotreatment with d-borneol and CDDP inhibited tumor growth in vivo and reduced CDDP-caused liver and kidney toxicity.

**Conclusions:**

d-Borneol increased the efficacy of cisplatin and reduced its toxicity. This compound has the potential to become a useful chemosensitizer for drug-resistance NSCLC.

**Supplementary Information:**

The online version contains supplementary material available at 10.1186/s11658-022-00362-4.

## Introduction

Lung cancer is a malignancy with high morbidity and mortality rates [1, 2]. Non-small cell lung cancer (NSCLC) is the most common form, accounting for about 85% of all occurrences [3]. Progress has been made in NSCLC treatment using surgery, chemotherapy, radiation, and molecularly targeted therapy, yet 5-year overall survival rate remains poor [4, 5]. Platinum-based chemotherapy is recommended as a first-line therapeutic option. Cisplatin (CDDP) is widely used clinically [6, 7], and is a key drug for treating multiple human cancers, including NSCLC. The drug acts by damaging DNA and inducing apoptosis [8, 9]. However, acquired resistance inexorably develops after a period of treatment and remains a major limitation to clinical application [10–12]. Synergistic combinations of drugs enhance therapeutic efficacy and are often used to overcome drug resistance. Thus, new and safe combinations to improve NSCLC cell sensitivity to CDDP are critical to successful treatment.

Combinations of agents derived from natural products with antitumor drugs have gained attention mainly owing to their ability to prevent cancer development and progression by inhibiting proliferation, differentiation, metastasis, and angiogenesis, or by inducing apoptosis and regulating autophagy and the cell cycle. These combinations aim to improve therapeutic efficacy, decrease the risk of adverse side effects and toxicity, and overcome the acquired drug resistance [13–16].

Borneol, a monoterpenoid isolated from several medicinal plants, including *Blumea blasamifera* (L.) DC. and *Cinnamomum camphora* (L.) Presl, is a characteristic traditional Chinese medicine (TCM) resuscitation agent. The compound can awaken and refresh and has been used for cardiovascular and cerebrovascular diseases for thousands of years. The Chinese Pharmacopoeia separates borneol into three categories: natural borneol (dextrorotatory borneol, d-borneol), levorotatory borneol (l-borneol), and synthetic borneol (SB), to reflect differences among sources [17]. Borneol is widely regarded as a messenger drug capable of delivering main effective drugs in the prescription to the target site to improve therapeutic efficacy. Borneol can enhance drug penetration across different physiologic barriers, such as the blood–brain barrier (BBB) [18], cornea [19], skin [20], and mucous membrane [21]. This property encourages increased blood concentrations and bioavailability of other drugs. d-Borneol seems safer than SB [22]. Further, borneol may prevent drug efflux by suppressing P-glycoprotein (P-gp) function and acting as a chemotherapeutic sensitizer to promote therapeutic efficacy [23]. For example, borneol sensitizes cancer cells to chemotherapeutic drugs, such as cisplatin [24], curcumin [25], paclitaxel [26], and doxorubicin [27], and enhances the effects of therapies for glioma, melanoma, hepatocellular carcinoma, and esophageal squamous cell carcinoma. However, the impact of d-borneol on NSCLC cells and its ability to stimulate chemosensitivity to CDDP in resistant cells is not well studied, and underlying processes remain unclear. Therefore, we initially selected an NSCLC cell line sensitive to d-borneol and established optimum concentrations and exposure durations. We used the corresponding cisplatin-resistant cell line, H460/CDDP, to investigate synergy between d-borneol and CDDP on proliferation, cell cycle progression, and apoptosis.

RNA sequencing (RNA-seq) is an approach for whole-transcriptome sequencing to analyze key factors of d-borneol-induced sensitization. We focused on cyclinA2/D3-CDK2/6 signaling pathway-related cell cycle arrest, the Bax/Bcl-2/caspase3 pathway associated with apoptosis, and P-gp function to attempt to explain mechanisms of synergy between d-borneol and CDDP. Lastly, we explored antitumor and major protective effects of d-borneol with CDDP in vivo.

## Materials and methods

### Cell lines and reagents

Human lung cancer A549 (CAT: FH0045) (Additional file 1) and H460 (CAT: FH0050) (Additional file 2) cells were kindly provided by Dr. Cao (Chengdu University of Traditional Chinese Medicine, Chengdu, China), and H460/CDDP cells were purchased from FuHeng Biology (CAT: FH1207, FuHeng Cell Center, Shanghai, China). At 37 °C in a humidified environment with 5% CO_2_, A549, NCI-H460, and H460/CDDP cells were cultured in Roswell Park Memorial Institute (RPMI)-1640 medium (Gibco, Australia) with 10% fetal bovine serum (FBS; Gibco, Australia) and 1% penicillin/streptomycin solution (Gibco, Australia) added. CDDP was obtained from Sigma-Aldrich (St. Quentin Fallavier, France). d-Borneol (#T5734) with purity (99.6%) was purchased from Shanghai Topscience Co., Ltd. DMSO (D8371) was purchased from Solarbio Science & Technology Co., Ltd. MTT reagent was purchased from BioFroxx. RT EasyTM I and Real Time PCR Easy SYBR Green One-Step Kit were purchased from Foregene Co., Ltd. Anti-Bax (T40051), Bcl-2 (T40056), and caspase-3 (M005851) were purchased from Abmart (Shanghai, China). Anti-cyclinA2 (ab181591), CDK2 (ab32147), and p27 (ab32034) were purchased from Abcam (Paris, France). Anti-p21 (AF6290), cyclinD3 (AF6251), CDK6 (DF6448), P-gp (AF5185) and tubulin (AF7011) were obtained from Affinity Biosciences.

### Determination of borneol type and cell line

The cytotoxicity of borneol on H460 and A549 cells was determined by MTT. Cells were seeded at a density of 5 × 10^3^ cells per well in 96-well plates and incubated for 24 h or 48 h. Then, cells were treated with different doses of d-borneol, l-borneol, and SB (0, 1, 2, 4, 8, 16, and 32 μg/ml) prepared in DMSO and diluted in RPMI-1640 with 1% FBS. After 24 h or 48 h, 20 μl MTT reagent was added to each well. Then, the reagent was removed after incubation for 4 h at 37 °C, and 100 μl DMSO was added. Absorbance at 570 nm was measured by Bio-Tek microplate reader after incubation for 10 min (BioTek, Winooski, VT, USA). Cell viability = (treated viable cells)/(control viable cells) × 100%.

### Cell viability assays of combined treatment

To evaluate the concentration of CDDP, H460 and H460/CDDP cells (5 × 10^3^ per well) were seeded in a 96-well plate and treated with CDDP (0.625–160 μg/ml) for 24 h. Then H460/CDDP cells were exposed to CDDP (0.625–160 μg/ml) for 24 h and 48 h to determine the suitable time. Next H460/CDDP cells was exposed to three borneol forms (0.5–16 μg/ml) for 24 h to determine the concentration and type of borneol. Finally for combined treatment, H460/CDDP cells were pretreated with d-borneol (0.5, 1, 2, and 4 μg/ml) for 12 h and co-incubated with CDDP (10 μg/ml) for 24 h. MTT assay, as described in “Determination of borneol type and cell line” section, was used to determine cell viability. The morphology of the cells was observed and photographed by inversion fluorescence microscopy (Leica DMI3000B, Germany).

### Evaluation of P-gp function assay

H460/CDDP cells were incubated with CDDP (10 μg/ml) plus d-borneol (0.5, 1, 2, and 4 μg/ml) for 24 h. The cells were resuspended in the medium, stained with rhodamine 123 (Rho 123) solution (Beyotime Biotechnology, Shanghai, China), and incubated for 30 min at 37 °C in 5% CO_2_. The cells were centrifuged for 5 min at 2000 rpm, then resuspended and incubated for 120 min after being washed twice with medium. Then the cells were centrifuged again and washed twice with PBS. A fluorescence spectrophotometer was used to measure the fluorescence of Rho 123 with emission wavelength of 529 nm and excitation wavelength of 507 nm (BioTek, Winooski, VT, USA).

### Scratch-wound healing recovery assays

A monolayer of H460/CDDP cells was scratched with the tip of a 100 μl pipette for the wound healing experiment, and the floating cells were rinsed off with PBS. Under a microscope, the gap widths were measured. After that, the cells were treated with different doses of CDDP (10 μg/ml) and d-borneol (0.5, 1, 2, and 4 μg/ml), and photographs of the wounds were taken in four random areas by microscope (Leica, Germany) at 0 h and 24 h. ImageJ was used to measure the scratch areas.

### Colony-formation assay

H460/CDDP cells were planted at a density of 2 × 10^5^ cells per well in six-well plates. The cells were next treated with CDDP (10 μg/ml) for 24 h, or with CDDP (10 μg/ml) with d-borneol (0.5, 1, 2, and 4 μg/ml) for 24 h. Cells were then digested with trypsin, and 500 cells were reseeded onto six-well plates and cultured for a further 8–10 days. Finally, crystal violet staining solution was used to stain the colonies (Beyotime Biotechnology, Shanghai, China). The number of colonies was quantified by ImageJ. The proportion of control was used to normalize the results.

### Flow cytometry cell cycle analysis

H460/CDDP cells were logarithmically cultivated and seeded into 6-cm dishes at a density of 2 × 10^4^ cells/ml. After treatment with the combination of d-borneol (0.5, 1, 2, and 4 μg/ml) and CDDP (10 μg/ml) for 24 h, H460/CDDP cells were collected, washed with PBS, and fixed with ethanol for 24 h. Then the cells were stained with propidium iodide (PI) for 30 min and protected from light. Flow cytometry was utilized to detect changes in cell cycle distribution (BD Biosciences, CA, USA) and analyzed by FlowJo V10 software [28].

### Apoptosis analysis by flow cytometry

Cells were treated with CDDP (10 μg/ml), or CDDP (10 μg/ml) plus d-borneol (0.5, 1, 2, and 4 μg/ml), for 24 h. H460/CDDP cells were harvested, washed, and stained with 5 μl FITC-annexin V and 5 μl PI (Keygen, Nanjing, China) according to the manufacturer’s protocol. The cells were incubated in the dark for 15 min at room temperature. The degree of cell apoptosis was assessed by flow cytometry (BD Biosciences, CA, USA).

### Hoechst 33258 staining

Hoechst 33258 (Beyotime Biotechnology, Shanghai, China) was used to evaluate the apoptosis in H460/CDDP cells after treatment with CDDP or d-borneol plus CDDP. In brief, cells were fixed with paraformaldehyde–glutaraldehyde fixing solution for 30 min. After being washed in PBS, the cells were further stained with Hoechst 33258 for 20 min at room temperature. The cells were then washed three times in PBS before being examined using a BSF-40 fluorescence microscope (Leica, Germany), and the level of cell apoptosis in each group was calculated.

### Mouse xenograft assays

Male Balb/c nude mice (4-week-old) were bought from SPF (Beijing) Biotechnology Co., Ltd. and kept in the Experimental Animal Research Center of Chengdu University of TCM. Mice were maintained in a temperature-controlled facility of 22 ± 2 °C with a 12-h/12-h light/dark cycle and a relative humidity of 55 ± 5% in specific pathogen-free facility. All mice were given free access to water and food. After 1 week of adaptable feeding, H460/CDDP cells (5 × 10^6^ cells in 0.1 ml phosphate-buffered saline) were subcutaneously injected into the right dorsal flank of male BALB/c nude mice. When tumor formation was confirmed, the mice were randomly divided into the following four treatment groups: a control group (Con, saline only, *n* = 6), a vehicle group (Vehicle, 2% tween), a d-borneol group (Bor, 200 mg/kg, *n* = 6), a CDDP group (CDDP, 3 mg/kg, *n* = 6), and a combination treatment group (CDDP plus d-borneol, CDDP + Bor). The mice in the CDDP group were injected with CDDP (3 mg/kg) every 3 days. The mice in the CDDP + Bor group were treated with 200 mg/kg of d-borneol and 3 mg/kg of CDDP. Tumor volume and body weight were recorded every 2 days after tumor injection. At the end of the test, the mice were sacrificed, and the tumors were harvested.

### RNA sequencing

Total RNA was prepared for cDNA libraries using protocol provided by Oxford Nanopore Technologies (ONT). Briefly, SuperScript IV First-Strand Synthesis System (Invitrogen) was used for full-length mRNA reverse transcription following cDNA PCR for 14 circles with LongAmp Tag (NEB). The PCR products were then subjected to formalin-fixed paraffin-embedded (FFPE) DNA repair and end-repair (NEB) steps and following adaptor ligation using T4 DNA ligase (NEB). Agencourt XP beads were used for DNA purification according to ONT protocol. The libraries were sequenced on PromethION platform at Biomarker Technology Company (Beijing, China).

### RNA extraction and quantitative real-time PCR

Total RNA was extracted using TRIzol reagent (Invitrogen, Carlsbad, CA, USA). The optical density (OD) at 260/280 nm was measured using a nucleic acid/protein analyzer for RNA purity testing, and then transformed to cDNA. The following were the RT-PCR reaction conditions: 95 °C for 10 min, 40 cycles of 15 s at 95 °C, and 30 s at 60 °C (Bio-Rad, USA). The primer sequences are presented in Table 1. The internal reference gene (GAPDH) was used to adjust the relative expression levels using the fluorescence quantitative analysis technique of 2^−ΔΔCt^ method.

**Table 1:** Primer sequences for real-time PCR assays

Name	Forward primer sequence (5′–3′)	Reverse primer sequence (5′–3′)
GAPDH	CAGGAGGCATTGCTGATGAT	GAAGGCTGGGGCTCATTT
ABCB1	GATTGCTCACCGCCTGTCCAC	CGTGCCATGCTCCTTGACTCTG
CCNA2	GCCAGACATCACTAACAGTATGAGAGC	GCACTGACATGGAAGACAGGAACC
cyclinD3	TGCCACAGATGTGAAGTTCATT	CAGTCCGGGTCACACTTGAT
CDK2	ATGAAGATGGACGGAGCTTGTTATCG	CTGGCTTGGTCACATCCTGGAAG
CDK6	CGAACAGACAGAGAAACCAAAC	CTCGGTGTGAATGAAGAAAGTC
p21	CATGTGGACCTGTCACTGTCTTGTA	GAAGATCAGCCGGCGTTG
p27	CTAACTCTGAGGACACGCATTT	TTGAGTAGAAGAATCGTCGGTT
Bax	GATGCGTCCACCAAGAAGCTGAG	CACGGCGGCAATCATCCTCTG
Bcl-2	TCGCCCTGTGGATGACTGAGTAC	TCAGAGACAGCCAGGAGAAATCAAAC
Caspase-3	GTGGAGGCCGACTTCTTGTATGC	TGGCACAAAGCGACTGGATGAAC

### Western blot analysis

H460/CDDP cells were treated with CDDP (10 μg/ml) plus d-borneol (2 μg/ml) for 24 h before protein extraction with radioimmunoprecipitation assay (RIPA) buffer supplemented with a complete phosphatase and protease inhibitor cocktail (Beyotime Biotechnology, Shanghai, China). Protein quantification was performed by BCA assay kit (Beyotime Biotechnology, Shanghai, China). SDS–PAGE separated the proteins, which were then transferred to PVDF (Millipore Corporation, Billerica, USA). The membranes were blocked with 5% skim milk for 2 h at room temperature and then washed with TBST. Then the membranes were incubated with the following primary antibodies overnight at 4 °C: anti-P-gp (1:1000), anti-cyclin A2 (1:2000), anti-cyclin D3 (1:1000), anti-CDK2 (1:5000), anti-CDK6 (1:1000), anti-p21 (1:1000), anti-p27 (1:5000), anti-Bax (1:1000), anti-Bcl-2 (1:1000), and anti-caspase-3 (1:1000). Appropriate secondary antibodies conjugated to horseradish peroxidase were used for 1 h at room temperature before exposure to enhanced chemiluminescence (ECL). Polyclonal rabbit tubulin (1:2000) was used as loading control. The relative density of each protein band was normalized to tubulin. A signal was acquired with a ChemiDocTMXRS + imaging system (Bio-Rad, Marnes-la-Coquette, France), and blots were analyzed with Image Lab software (Bio-Rad) and ImageJ.

### Histological examination

Tumor tissue was fixed in 10% formalin for 24 h, embedded in paraffin, and sliced every 5 μm. After dewaxing in xylene, the tissue was dehydrated in decreasing concentrations of alcohol and stained with hematoxylin and eosin (H&E). Then, the representative areas were captured by inversion fluorescence microscopy at 200× and 400× magnification (Leica DMI3000B, Germany).

### Immunohistochemistry

Tumor tissues were fixed, paraffin-embedded, and sectioned. The deparaffinized and rehydrated sections were subjected to antigen retrieval using sodium citrate buffer. After incubation with 10% normal goat serum for 1 h, the sections were incubated with primary antibody overnight at 4 °C. The subsequent procedures were performed according to the manufacturer’s instructions. Then, the representative areas were captured by inversion fluorescence microscopy at 200× and 400× magnification (Leica DMI3000B, Germany).

### Biochemical assay

The blood samples were centrifuged at 3000 rpm (10 min, 4 °C) to collect plasma, and then levels of albumin (ALB), total protein (TP), lactate dehydrogenase (LDH), alanine aminotransferase (ALT), aspartate aminotransferase (AST), blood urea nitrogen (BUN), and creatinine (CRE) were determined by Roche Automatic biochemical analyzer (cobas c311) according to the respective kits purchased from Roche Diagnostics GmbH (Shanghai, China).

### Statistical analysis

Statistical analysis was performed using SPSS statistical package. When there were only two groups, the differences were examined via *t*-test, and when there were more than two groups, they were assessed by one-way analysis of variance (ANOVA). Data are expressed as mean ± standard deviation (SD). *,^#^ denote *P* < 0.05.

## Results

### Cytotoxicity of the three borneol forms on NSCLC cells

**Fig. 1:**
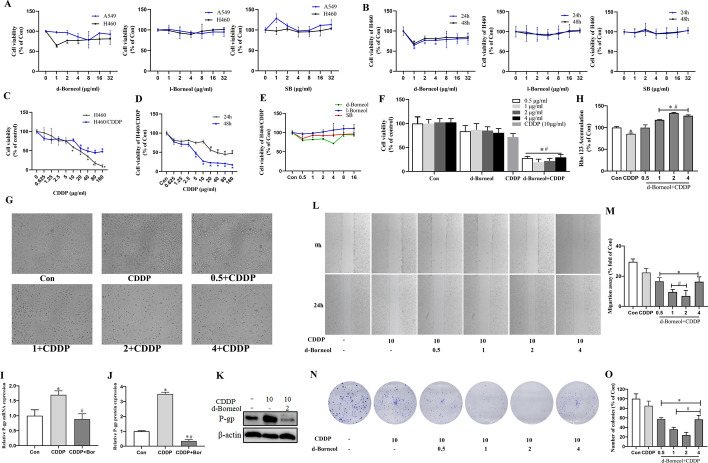
Cell viability of different NSCLC cells treated with three borneol forms and cotreatment with d-borneol and CDDP, and effect on cell proliferation, P-gp function, and cell migration in H460/CDDP cells. **A** A549 and H460 cells cultured with different concentrations of d-borneol, l-borneol, and SB for 24 h; **B** H460 was treated with d-borneol, l-borneol, and SB for 24 h and 48 h, and cell viability was determined by MTT; **C** cell viability of H460 and H460/CDDP cells treated with different concentrations of CDDP for 24 h; **D** cell viability of CDDP on H460/CDDP cells for 24 h and 48 h; **E** cytotoxicity of the three borneol forms on H460/CDDP cells; **F**, **G** cell viability (**F**) and cell morphology (**G**) of H460/CDDP cells treated with CDDP (10 μg/ml), or d-borneol (0.5, 1, 2, and 4 μg/ml) + CDDP (10 μg/ml) for 24 h; **H** determination of P-gp function after treatment with CDDP, CDDP plus d-borneol with different concentrations for 24 h; **I**–**K** mRNA expression (**I**) and protein expression (**J**, **K**) of P-gp; **L**, **M** cell migration of H460/CDDP cells; **N** representative photographs showing colony formation after CDDP cotreatment with d-borneol in H460/CDDP cells; **O** calculation of statistical differences. All data are presented as mean ± SD (*n* = 3, *P < 0.05, compared with Con; ^#^*P* < 0.05, compared with CDDP group)

A549 and H460 cells were treated with varying doses of three borneol forms for 24 h. Cell viability was assessed by MTT assay. A549 and H460 cells were treated with l-borneol and SB at concentrations of 1–32 μg/ml for 24 h, and no apparent cytotoxicity was seen (Fig. 1A). However, d-borneol at concentrations ranging from 1 to 4 μg/ml significantly inhibited H460 cell proliferation, with the greatest inhibition at 1 μg/ml. Further, no significant cytotoxicity was seen between 24 and 48 h (Fig. 1B). Therefore, d-borneol concentrations of 0.5, 1, 2, and 4 μg/ml and 24 h were chosen as suitable concentrations and duration time, respectively, for further experiments.

### d-Borneol potentiates the cytotoxicity of CDDP

H460 and H460/CDDP cells were treated with different doses of CDDP for 24 h to identify the suitable concentration (Fig. 1C). CDDP reduced the viability of H460 cells in a dose-dependent manner, with doses ranging from 0.625 to 160 μg/ml. In contrast, H460/CDDP cells did not exhibit dose-dependent inhibition. H460 cell viability was reduced by 48.18% at a CDDP concentration of 20 μg/ml. A concentration of 40 μg/ml was required to achieve such toxicity for H460/CDDP cells (Fig. 1C). Thus, H460/CDDP cells were relatively resistant to CDDP, especially at concentrations below 10 μg/ml. CDDP (10 μg/ml) was chosen as a concentration for subsequent synergy studies. To confirm the suitable exposure time of CDDP, the cell viability of CDDP on H460/CDDP cells for 24 h and 48 h was also measured. Compared with 24 h, the viability of H460/CDDP cells was inhibited overly after 48 h (Fig. 1D). Thus, 24 h was selected as a suitable exposure time for continuing experiments. Furthermore, d-borneol was much more active against H460/CDDP cells than l-borneol and SB, especially in the concentration range of 0.5 μg/ml to 4 μg/ml (Fig. 1E). On the basis of these findings, CDDP (10 μg/ml), d-borneol (0.5–4 μg/ml), and 24 h were thus selected as the appropriate exposure conditions.

We found that exposure to d-borneol alone caused slight cell growth suppression. In addition, exposure to CDDP (10 μg/ml) alone and in combination with d-borneol (0.5, 1, 2, and 4 μg/ml) inhibited viability of H460/CDDP cells by 28.21%, 72.27%, 80.61%, 78.08%, and 70.46%, respectively (Fig. 1F). Exposure to d-borneol thus increased the sensitivity of H460/CDDP cells to CDDP, especially at concentrations of 1 and 2 μg/ml. Further, the cells in the Con group adhered well with clear and smooth edges and showed few round cells after exposure to CDDP alone. More suspended cells and cell fragments were found after combined exposure, especially at a d-borneol concentration of 2 μg/ml (Fig. 1G).

### d-Borneol increases CDDP sensitivity by suppressing P-gp

P-gp-mediated drug efflux is key to chemoresistance in NSCLC. Rhodamine 123 (Rho 123) is a fluorescent dye eliminated from cells by P-gp; greater cellular accumulation of Rho123 is widely viewed as a sign of impaired P-gp-mediated efflux. P-gp functional analysis showed enhanced Rho 123 accumulation when CDDP exposure was combined with d-borneol in H460/CDDP cells. A d-borneol concentration of 2 μg/ml induced the greatest response (Fig. 1H). Further, we showed that mRNA and protein levels of P-gp were upregulated after exposure to CDDP alone, indicating that resistance is accompanied by increased P-gp expression. Conversely, expression was significantly downregulated in cells exposed to CDDP and d-borneol (CDDP + Bor), suggesting that P-gp inhibition might be an important mechanism for d-borneol and CDDP synergy (Fig. 1I–K).

### d-Borneol combined with CDDP inhibits clone formation and migration

We examined the area between wound edges to assess d-borneol impacts on H460/CDDP cell migration. The treatment of d-borneol combined with CDDP displayed migration inhibitory ability in H460/CDDP cells compared with CDDP alone, especially at d-borneol concentrations of 1 and 2 μg/ml, with an index of migration of 9.53 ± 1.74% and 6.82 ± 3.72%, respectively, compared with the CDDP group (22.43 ± 2.76%) at 24 h (Fig. 1L, M). Additionally, the capacity of a single cell to develop into a colony was tested by colony formation experiments. H460/CDDP cells were cotreated with different doses of d-borneol (0.5, 1, 2, 4 μg/ml), and the number of colonies was determined by violet-blue staining. H460/CDDP cells cotreated with CDDP and d-borneol showed significantly reduced colony formation compared with CDDP treatment alone, especially at a d-borneol concentration of 2 μg/ml (Fig. 1N, O). The data indicating that combined d-borneol increased CDDP sensitivity are thus reflected in reduced cell growth and migration, especially at 2 μg/ml d-borneol.

### d-Borneol increased CDDP sensitivity by enhancing cell apoptosis

**Fig. 2:**
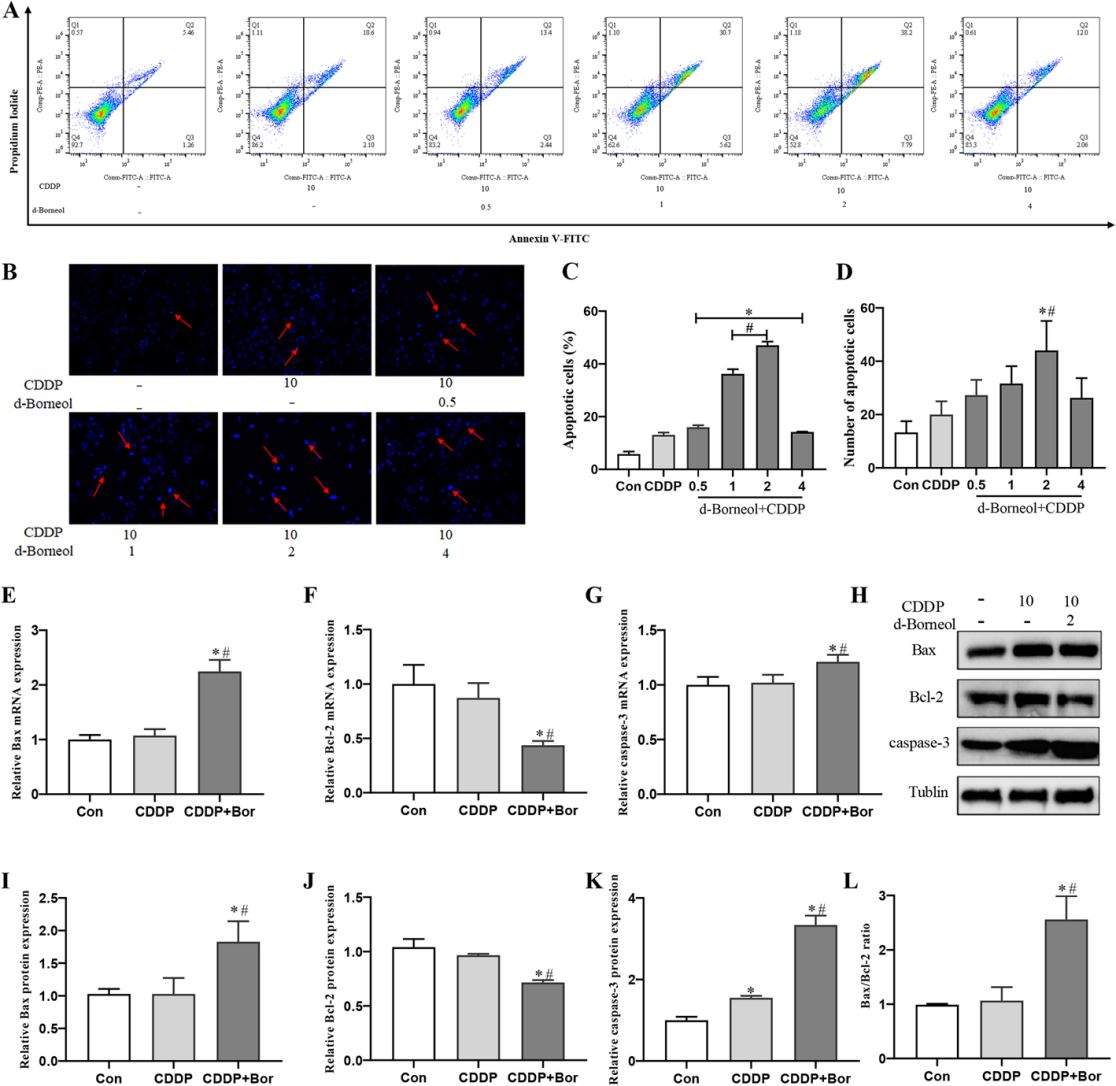
d-Borneol induced cell apoptosis in H460/CDDP cells. **A**, **C** Flow cytometry showing the apoptotic cell percentages in each group; **B**, **D** representative picture of H460/CDDP cells Hoechst staining in each group and quantitative analysis; **E**–**G** mRNA expression of Bax (**E**), Bcl-2 (**F**), and caspase-3 (**G**); **H**–**K** protein expression of Bax, Bcl-2, and caspase-3 and their relative statistical analysis; **l** ratio of Bax/Bcl-2. All data are presented as mean ± SD (*n* = 3, *P < 0.05, compared with Con; ^#^*P* < 0.05, compared with CDDP group)

The percentage of apoptotic cells in H460/CDDP cells was measured by annexin V/PI double labeling. There was no significant difference in percentage of apoptotic cells between the CDDP group and the Con group, while significantly more apoptotic cells were observed in synergetic therapy of d-borneol and CDDP. The population of apoptotic cells in the CDDP group was 13.07 ± 0.93%, and it was elevated to 36.26 ± 1.73% and 47.03 ± 1.46% when cotreated with d-borneol (1 and 2 μg/ml), respectively (Fig. 2A, C). Further, Hoechst staining showed faint homogeneous blue nuclei after exposure to CDDP alone. Brighter blue staining with chromatin condensation and nuclear fragmentation was observed with combined treatment, indicating activation of apoptotic processes (Fig. 2B, D). These results revealed that d-borneol acts synergistically with CDDP to stimulate apoptosis, especially at a concentration of 2 μg/ml.

### d-Borneol induces apoptosis via regulating Bax/Bcl-2/caspase-3 signaling

To further explore the apoptosis mechanism of d-borneol and CDDP on H460/CDDP cells, apoptosis-related protein and gene expression was determined by western blotting assay and real-time PCR, respectively. Expression of pro-apoptosis genes, *Bax* and *caspase-3*, after cotreatment with d-borneol and CDDP was upregulated compared with treatment with CDDP alone; expression of anti-apoptotic *Bcl-2* was dramatically downregulated (Fig. 2E–G). Moreover, similar changes were found for protein expression of Bax (Fig. 2H, I), Bcl-2 (Fig. 2H, J), and caspase-3 (Fig. 2H, K). Notably, a significant increase in Bax/Bcl-2 expression ratio was seen after cotreatment (2.55 ± 0.42) compared with CDDP alone (1.07 ± 0.25) (Fig. 2L). d-Borneol thus induced apoptosis by modulating Bax/Bcl-2/caspase3 signaling in H460/CDDP cells to sensitize cells to CDDP synergistically.

### d-Borneol increased CDDP sensitivity by arresting the cell cycle

**Fig. 3:**
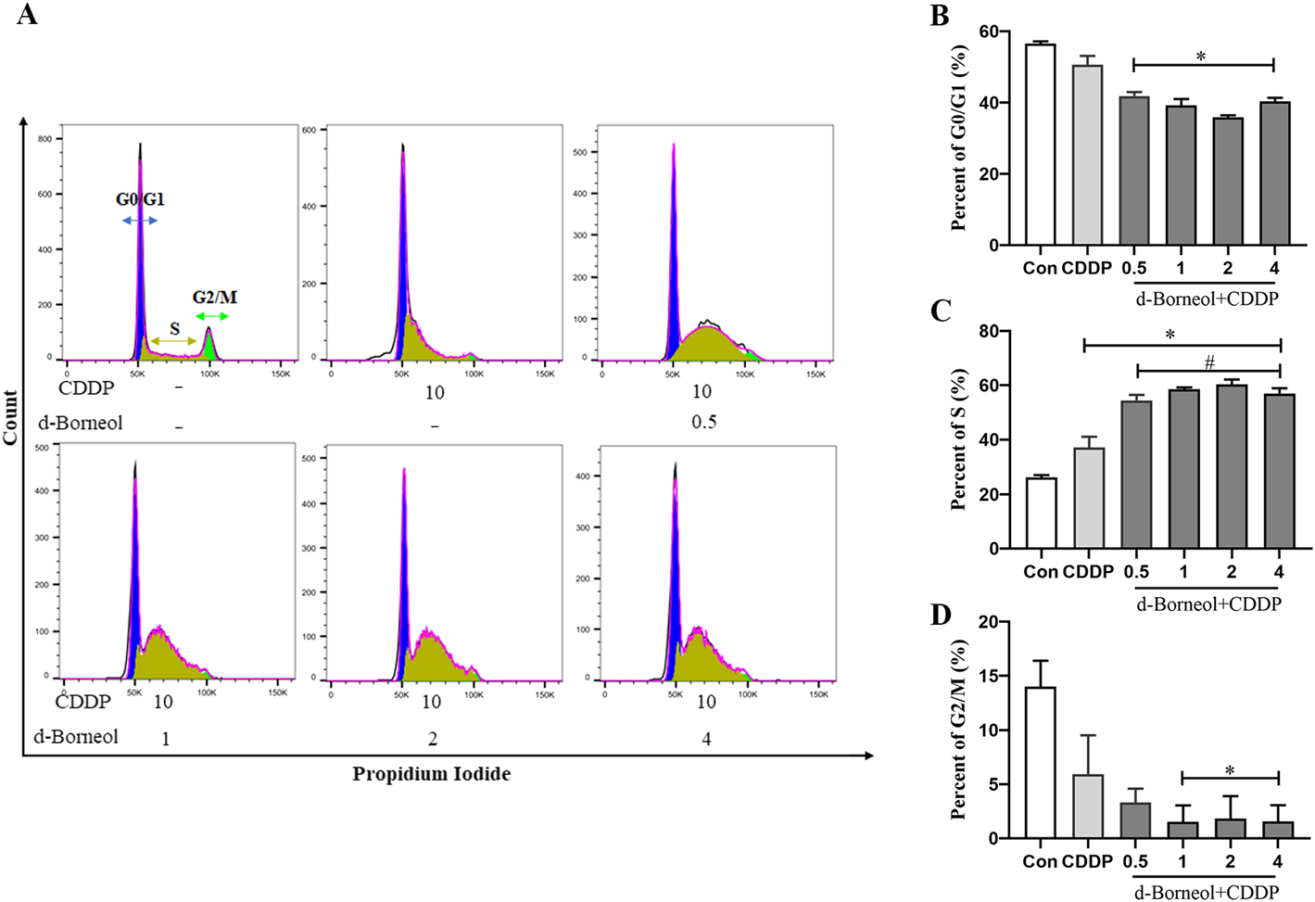
d-Borneol arrested cell cycle at S phase. **A** Cell cycle distribution of H460/CDDP cells detected by flow cytometry; **B**–**D** histogram and relative statistical analysis of H460/CDDP cells in G0/G1 phase, S phase, and G2/M phase. All data are presented as mean ± SD (*n* = 3, *P < 0.05, compared with Con; ^#^*P* < 0.05, compared with CDDP group)

Flow cytometry was performed to analyze the cell cycle distribution after 24 h when cotreated with d-borneol (0.5, 1, 2, and 4 μg/ml) and CDDP (10 μg/ml). As shown in Fig. 3A–D, compared with the Con group, CDDP showed a decreased tendency of G0/G1-phase percentage but elevated the percentage of S phase significantly. Cotreatment with d-borneol increased the percent of cells in S phase from 37.1% to 60.33% at a concentration of 2 μg/ml, indicating that d-borneol can enhance sensitivity of cells to CDDP via S-phase arrest.

### RNA-seq analysis supports the involvement of cell cycle arrest

**Fig. 4:**
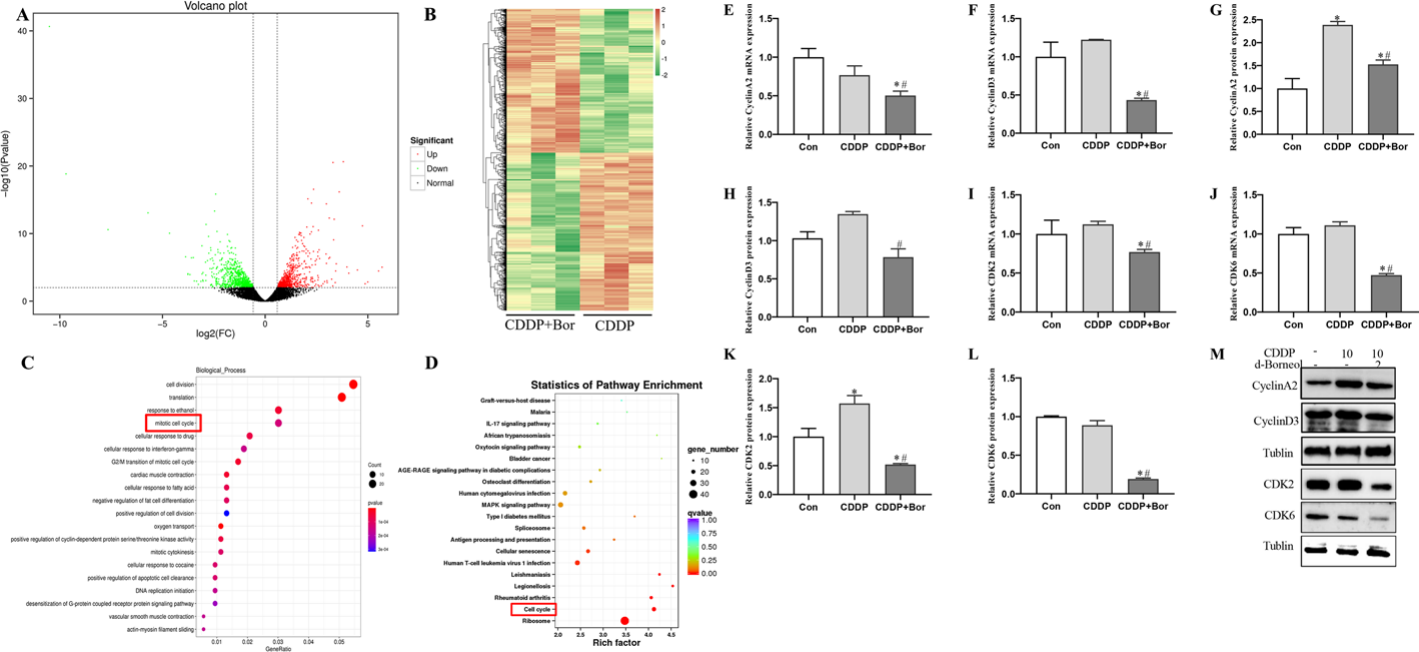
RNA-seq revealed cotreatment with d-borneol and CDDP enriched in cell cycle. **A** Volcano plot displaying the dysregulated genes between CDDP + Bor and CDDP groups; the red points represent significantly upregulated genes, and the green points represent significantly downregulated genes in CDDP + Bor; **B** heatmap showing differentially expressed genes in CDDP + Bor treatment groups compared with CDDP groups; **C** GO enrichment analysis of biological processes of downregulated genes in CDDP + Bor treated group; **D** KEGG pathway enrichment analysis of downregulated differentially expressed genes in CDDP + Bor group. Each group has three biological replicates; **E**–**H**, **M** mRNA expression level of cyclin A2 (**E**) and cyclin D3 (**F**) and protein level of cyclin A2 (**G**, **M**) and cyclin D3 (**H**, **M**); **I**, **J** mRNA expression level of CDK2 (**I**) and CDK6 (**J**); **K**–**M** western blotting and quantification analysis of CDK2 and CDK6. All data are presented as mean ± SD (*n* = 3, *P < 0.05, compared with Con; ^#^*P* < 0.05, compared with CDDP group)

Transcriptome sequencing (RNA-seq analysis) of full-length cDNA identified 1510 differentially expressed genes (DEGs) between CDDP group and CDDP + Bor group using fold change > 1.5 and FDR < 0.05 as screening criteria. These DEGs included 720 upregulated and 790 downregulated genes. A volcano map was generated to illustrate the overall distribution of DEGs, where red represents upregulated and green represents downregulated genes (Fig. 4A). Hierarchical clustering analysis further illustrates these genes (Fig. 4B).

GO and KEGG pathway enrichment analyses showed that downregulated genes were mainly enriched in cell-cycle-related pathways (red box) (Fig. 4C, D). These data prove that d-borneol cotreatment with CDDP suppresses NSCLC mainly through downregulating cell cycle processes, consistent with the previous flow cytometry results. Therefore, we hypothesized that cell cycle arrest may be a key determinant of cell death induced by d-borneol and CDDP.

### d-Borneol induced S-phase arrest via regulating cyclin/CDK complex signaling pathways

Three kinds of related molecules are involved in cell cycle regulation: cyclins, cyclin-dependent kinases (CDKs), and cyclin-dependent kinase inhibitor (CKI). Cyclin and CDKs tend to form the cyclin/CDK complexes that modulate cell cycle progression. Real-time PCR and western blotting analyses demonstrated the expression of genes and proteins related to S-phase processes, including cyclinA2, cyclinD3, CDK2, and CDK6. CyclinA2 mRNA (Fig. 4E), cyclinD3 mRNA (Fig. 4F), cyclinA2 protein (Fig. 4G, M), and cyclinD3 protein (Fig. 4H, M) expression were significantly downregulated in the CDDP + Bor group compared with the CDDP group. In addition, the CDDP + Bor group also decreased CDK2 and CDK6 mRNA (Fig. 4I, J) and protein (Fig. 4K–M) expression compared with CDDP alone.

### d-Borneol combined with CDDP upregulated expression of p27 and p21

**Fig. 5:**
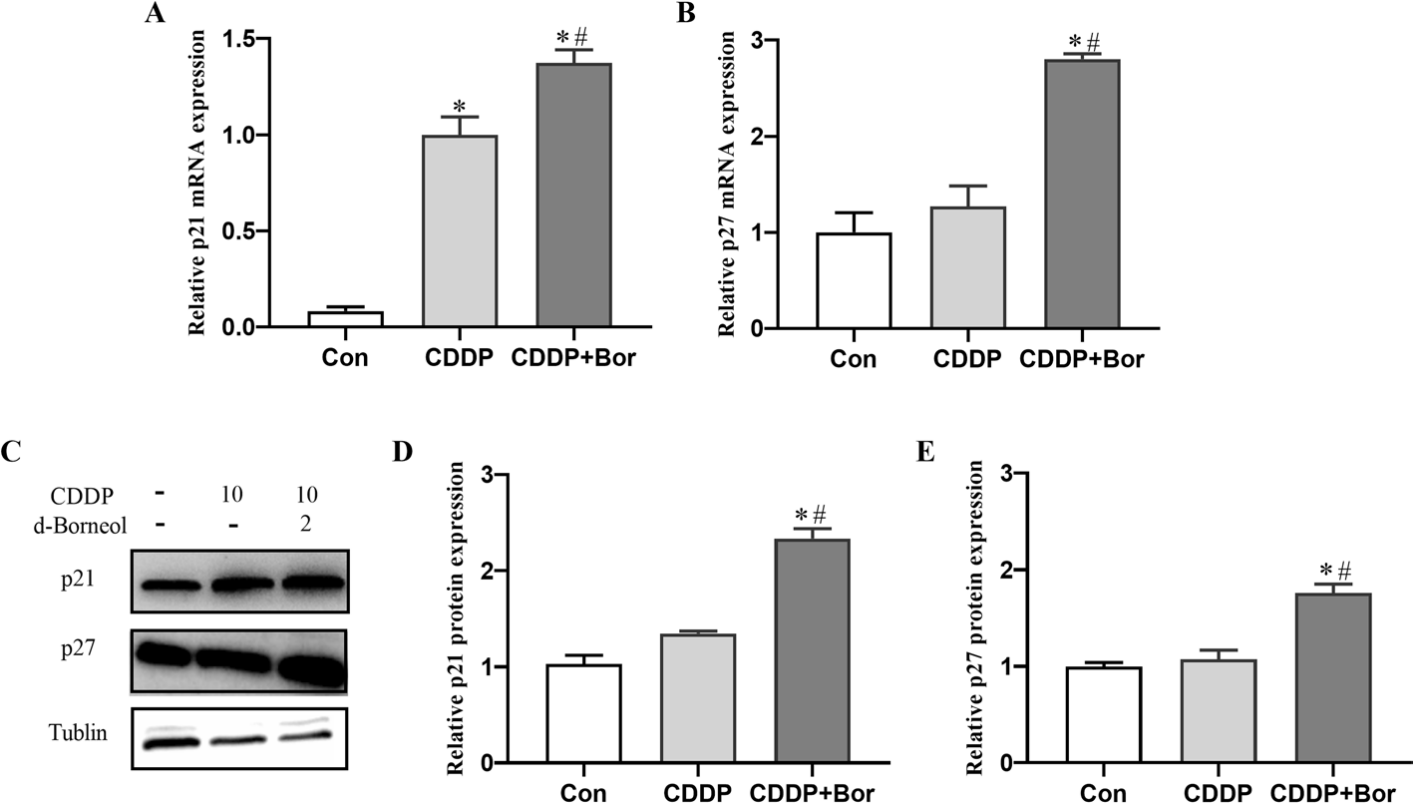
Expression of p27 and p21 in H460/CDDP cells. **A**, **B** mRNA expression level of p21 (**A**) and p27 (**B**) in each group; **C**–**E** protein expression of p21 and p27 and their quantification analysis. All data are presented as mean ± SD (*n* = 3, *P < 0.05, compared with Con; ^#^*P* < 0.05, compared with CDDP group)

CKIs, such as p21 and p27, play a crucial role in cell cycle progression as negative upstream regulators of CDKs and cyclins. Combined treatment elevated mRNA expression (Fig. 5A, B) and protein expression (Fig. 5C–E) of p21 and p27 significantly more than CDDP alone in H460/CDDP cells. These findings suggest that d-borneol induced S-phase arrest in H460/CDDP cells by upregulating p21 and p27 and downregulating cyclinA2/CDK2 and cyclinD3/CDK6 complexes.

### d-Borneol combined with CDDP suppresses NSCLC tumor growth in vivo

**Fig. 6:**
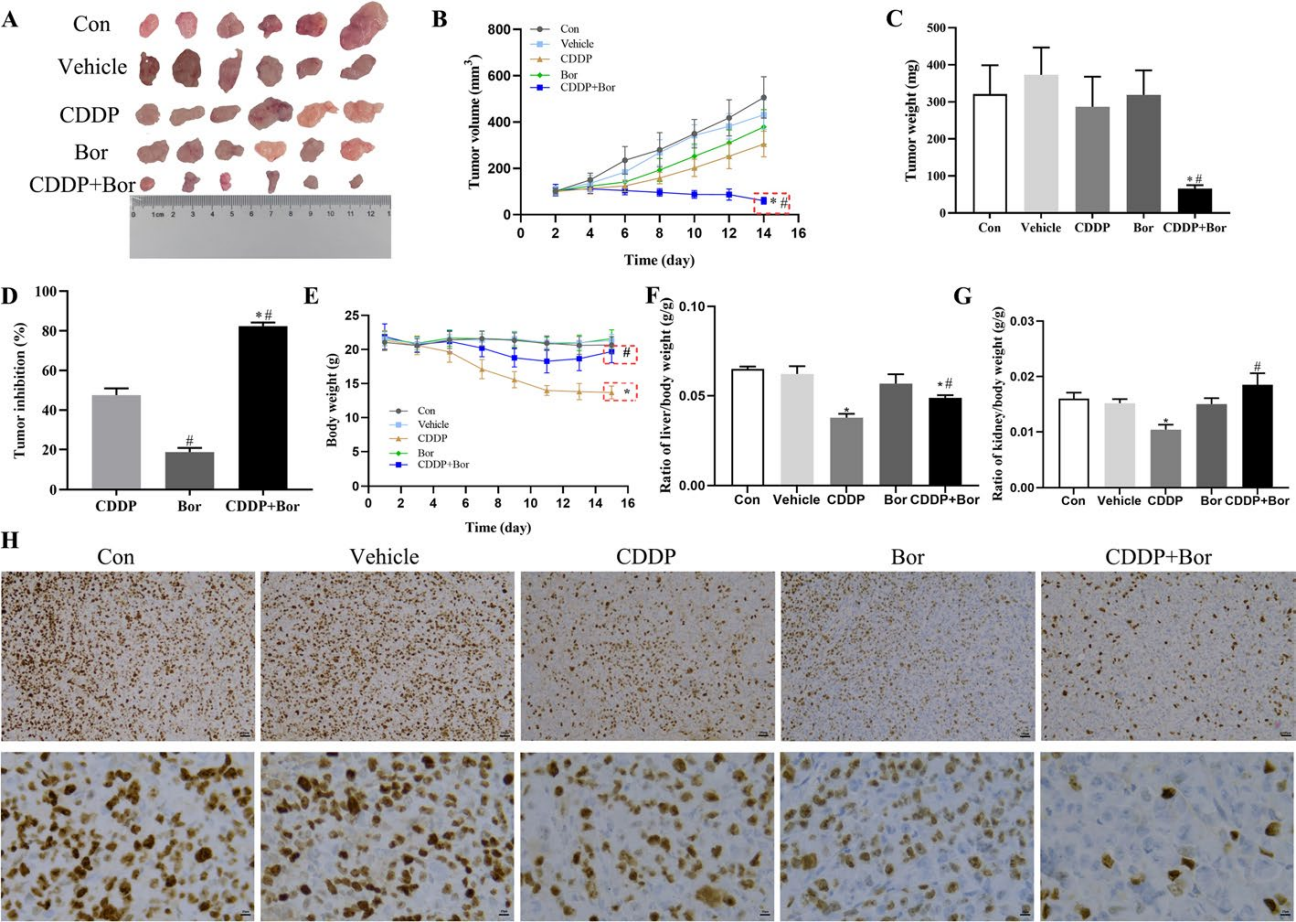
d-Borneol potentiates CDDP antitumor activities in vivo. **A** Representative photograph of tumors in different treatment groups; **B** tumor volume; **C** tumor weight; **D** tumor inhibition; **E** body weight; **F** organ index of liver; **G** organ index of kidney; **H** immunohistochemical staining of Ki67 from tumor tissues. Each value represent mean ± SD (*n* = 6, *P < 0.05, compared with Con, Vehicle, Bor group; ^#^*P* < 0.05, compared with CDDP group)

An H460/CDDP xenograft tumor model showed rapid tumor volume growth in untreated nude mice. CDDP treatment tended to inhibit tumor growth, but without a statistically significant impact. Conversely, combined treatment significantly inhibited tumor growth (Fig. 6A, B). Tumor weight data showed similar inhibition 14 days after combined treatment (Fig. 6C). Tumor growth inhibition was 82.35% for the combination of CDDP and d-borneol compared with 47.52% for CDDP alone (Fig. 6D).

We noted that treatment with d-borneol alone caused no notable body weight change, but the body weight of animals treated with CDDP was significantly lower than that of those in the Con group. After 14 days of treatment with the combination of d-borneol and CDDP, the CDDP + Bor group increased body weight significantly compared with the CDDP group, indicating that d-borneol potentiates the toxicity of cisplatin in vivo (Fig. 6E). Further, animals treated with CDDP exhibited decreased liver and kidney organ indices, and a restored effect was observed in animals treated with the combination of CDDP and d-borneol (Fig. 6F, G). Finally, immunohistochemical staining revealed significant downregulation of Ki-67 expression after d-borneol and CDDP treatment, suggesting that d-borneol might inhibit lung cancer growth (Fig. 6H). Overall, d-borneol and CDDP act synergistically to suppress tumor growth in vivo.

### Toxicity evaluation of d-borneol in combination with CDDP in vivo

**Fig. 7:**
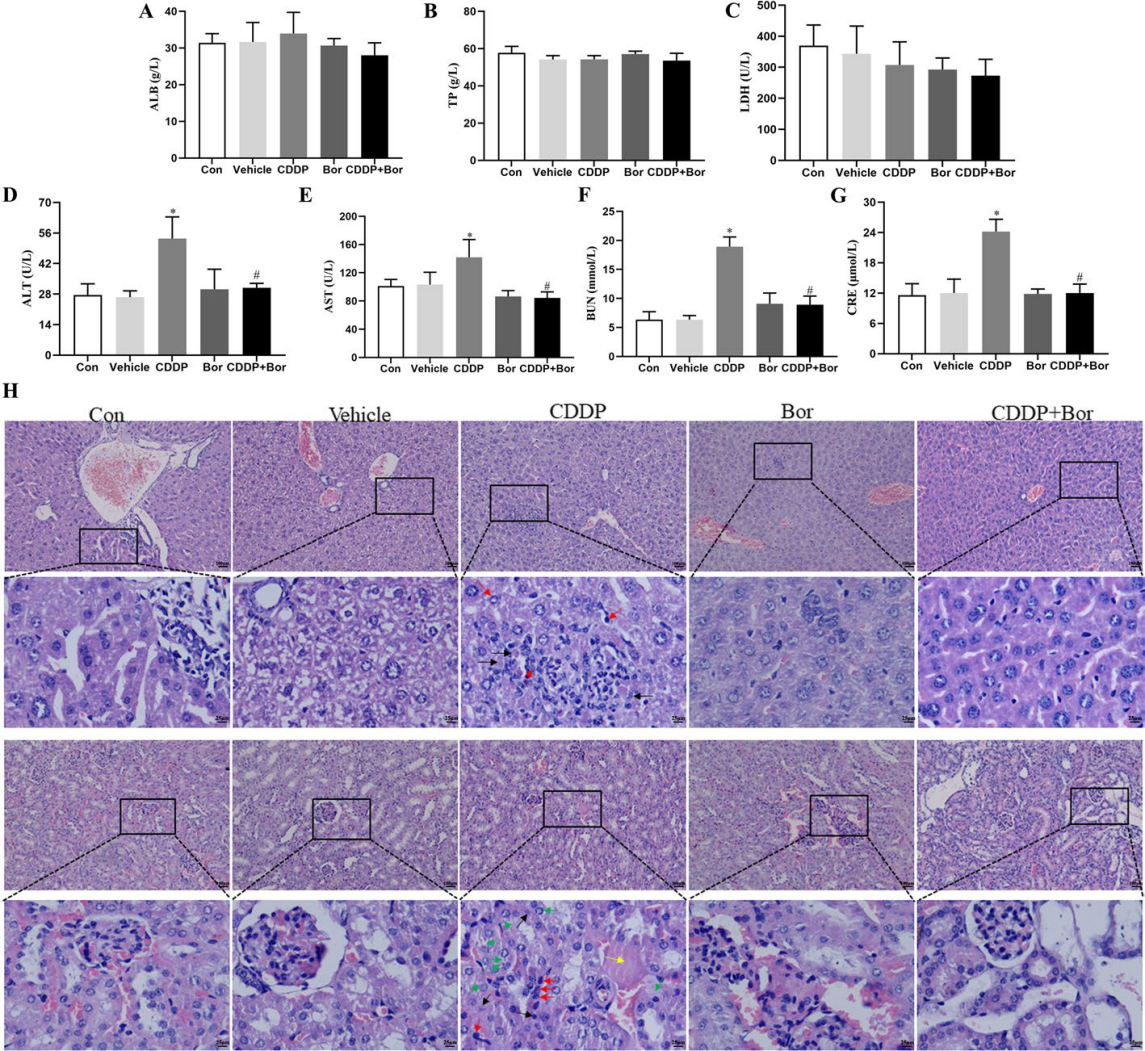
In vivo biotoxicity assessment. **A**–**G** Blood biochemistry data, including ALB (**A**), TP (**B**), LDH (**C)**, ALT (**D**), AST (**E**), BUN (**F**), and CRE (**G**). **H** H&E-stained results of liver and kidney. The pathological changes were observed under light microscope with ×100 and ×400 magnification. Red arrow represents inflammatory cells, black arrow represents necrosis, green arrow represents renal tubular epithelial edema cells, yellow arrow represents damaged casts. Each value represents means ± SD (*n* = 6, *P < 0.05, compared with Con, Vehicle, Bor group; ^#^*P* < 0.05, compared with CDDP group)

Although the combination therapy of d-borneol and CDDP shows substantial antitumor potential, ensuring the safety of combination therapy is crucial to the development of innovative chemosensitizers. Therefore, liver and kidney were sectioned and stained with H&E, and biochemical blood indicators, including ALB, TP, LDH, ALT, AST, BUN, and CRE, were analyzed. ALB (Fig. 7A), TP (Fig. 7B), and LDH (Fig. 7C) were not altered by any treatment. CDDP exposure induced a significant increase in ALT (Fig. 7D), AST (Fig. 7E), BUN (Fig. 7F), and CRE (Fig. 7G) levels; combination treatment decreased these markers effectively, suggesting that CDDP can cause liver and kidney toxicity, and d-borneol can alleviate these adverse effects. In addition, no histological abnormalities in liver or kidney were observed in the Con group. Still, increased inflammatory cells (red arrow) and necrosis (black arrow) were found in both liver and kidney tissue after intraperitoneal injection of cisplatin. In addition, renal tubular epithelial edematous cells (green) increased, and damaged casts (yellow) appeared in the lumen. When combined with d-borneol, inflammatory and edema cells were significantly decreased, necrotic cells were basically absent, and the cell structure was more regular, indicating that d-borneol ameliorated CDDP toxicity and protected functions of major organs (Fig. 7H).

## Discussion

Cisplatin-based chemotherapy is the principal approach to NSCLC treatment, but chemoresistance and adverse side effects limit its success for patients with advanced NSCLC [29]. Hence, developing chemosensitizers with high efficacy and low toxicity is of great significance to enhance the sensitivity to chemotherapeutic agents. Unique chemical structures, biological mechanisms, multiple target effects, ready applicability, and higher safety provide natural products with the potential for improving prevention and treatment of various diseases. Such activity may also increase efficacy and reduce side effects when combined with chemotherapeutic drugs [30].

Borneol, a traditional Chinese medicine, resuscitates and refreshes, and is usually used to treat cerebrovascular diseases. Natural borneol can boost the antitumor properties of selenocysteine [31] and curcumin [32] in hepatocellular carcinoma. However, whether borneol enhances sensitivity to chemotherapeutic drugs in NSCLC, and its underlying mechanism, is still unclear. Moreover, few studies compare the effects of three forms of borneol (d-borneol, l-borneol, and SB) in parallel on NSCLC cells. Therefore, this study is the first to investigate these compounds in lung cancer cells, identify the most sensitive cell lines and borneol forms, focus on the synergistic effects of borneol with CDDP, and systematically investigate chemosensitization mechanisms using RNA-seq analysis.

Multidrug resistance (MDR) usually manifests owing to the overexpression of P-gp, the main factor limiting response to chemotherapy in many cancers [33]. Inhibition of drug efflux mediated by P-gp might resensitize resistant cancer cells to chemotherapy [34]. Natural products are known to enhance the cytotoxicity to drug-resistant cells by physically inhibiting P-gp activity [35]. This action is consistent with our results, which indicate that d-borneol sensitized H460/CDDP cells to CDDP by inhibiting P-gp expression and function.

RNA-seq is a high-throughput sequencing technology with the advantages of rapid analysis and high resolution. RNA-seq analysis showed that the downregulated genes were mainly enriched in cell cycle. Arrest at any cycle phase will cause cell growth stagnation or death. The cell cycle is a continuous process and sensitive to disruption [36]. Further, altering the regulation of cell cycle progression is a crucial strategy for eliminating cancer cells [37, 38]. CDK kinase complexes are formed and activated by sequential activation and inactivation of CDK and cyclins at specific stages of the cell cycle. This sequential activation and inactivation is necessary for progress through the cell cycle [39]. Many natural products can block the cell cycle at different phases in various cancer cell lines. This block is caused by downregulating the expression of cyclin A, D, and E subunits and reducing the activity of cyclin/CDK [40–42]. Cyclin D, in particular, is associated with CDK4/6 and is the key regulator of G1-to-S transition [43]. S phase is the DNA replication stage, which is the key part of the entire cell cycle. Complexes formed by cyclin A/cyclin E and their respective kinase (CDK2) promote the progression through S phase, and downregulation of these cyclins and CDKs results in the accumulation of cells in S phase [44, 45]. The cyclin A/CDK2 complex affects both the beginning of DNA synthesis and the S-phase progression [46]. Flow cytometry showed that cotreatment with d-borneol and CDDP increased cell number in S phase but decreased G0/G1 phase, suggesting that d-borneol arrested the cell cycle at S phase. Further, western blotting showed that protein expression of CDK2, CDK6, cyclin A, and cyclin D decreased, consistent with the results of flow cytometry. Thus, d-borneol enhanced sensitivity to CDDP in H460/CDDP cells by modulating CDKs and cyclins associated with S-phase arrest.

CKI is an upstream regulator of CDKs and cyclins and is vital for cell cycle progression [47]. p21 and p27 are universal CKIs that physically bind to and inhibit functions of cyclin–CDK2, cyclin–CDK1, and cyclin–CDK4/6 complexes. This inhibition ultimately leads to the interruption of the cell cycle in G1 and S phases [48, 49]. Downregulation of p21 is linked to tumor differentiation, invasion, proliferation, and metastasis in several studies [50, 51]. Moreover, targeting p21 is a potential strategy for the treatment of tumors. Abnormal expression of p21 and p27 proteins is critical for the appearance of drug-resistant phenotypes following cancer therapy [52]. d-Borneol and CDDP together induced upregulated expression of mRNA and protein levels of p21 and p27 to levels that exceeded those seen for CDDP used alone. Similarly, Liu et al. [53] reported that the upregulation of p21 in lung cancer cells arrested the cell cycle, inhibited cell proliferation, and increased sensitivity to cisplatin. Taken together, we have shown that d-borneol combined with CDDP induced cell cycle arrest at S phase by upregulating p21 and p27 and suppressing cyclin A2/CDK2 and cyclin D3/CDK6 to enhance CDDP sensitivity in H460/CDDP cells through RNA-seq.

Cell apoptosis is critical for cancer prevention and therapy [54]. Apoptosis is mediated by two primary pathways: the mitochondria-driven apoptotic intrinsic pathway and the death receptor-induced extrinsic pathway [55]. p21 binds to Bcl-2 family proteins to release Bax proteins through the formation of a p21/Bcl-w complex, thus promoting cell apoptosis and inhibiting cell invasion [56]. The Bcl-2 family, which includes pro-apoptotic members (Bax, Bad, and Bak) and anti-apoptotic members (Bcl-2, Bcl-xL, and Bcl-B), plays a major role in the initiation of the intrinsic apoptotic pathway [57]. Caspases are a cysteine protease family and crucial effector molecules in caspase-dependent apoptosis. Activation of caspase-3 may initiate cell apoptosis [58]. Our results indicated that d-borneol and CDDP cotreatment promoted cell apoptosis and thus enhanced the sensitivity of CDDP to H460/CDDP cells via p21-mediated Bax/Bcl-2/caspase-3-activated intrinsic cell apoptosis.

To confirm the antitumor activity of d-borneol combined with CDDP in vivo, H460/CDDP cells were subcutaneously transplanted into nude mice. Combined treatment of d-borneol and CDDP after 14 days significantly inhibited tumor volume and weight while causing no obvious decrease in body weight, compared with the CDDP group. Interestingly, serum levels of ALT, AST, BUN, and CRE in the CDDP group were significantly increased, accompanied by obvious liver and kidney histopathological damage. Many inflammatory and necrotic cells were seen in liver and kidney tissue, edematous renal tubular epithelial cells were observed, and damaged casts appeared in kidney. However, no damage was observed in tissues from mice treated with CDDP and d-borneol. CDDP damaged in liver and kidney is thus ameliorated by combined treatment. Thus, animals that received both agents showed reduced tumor growth, and also less liver and kidney toxicity. This finding demonstrates the advantage of the combination treatment for increasing efficacy and reducing toxicity.

## Conclusions

**Fig. 8:**
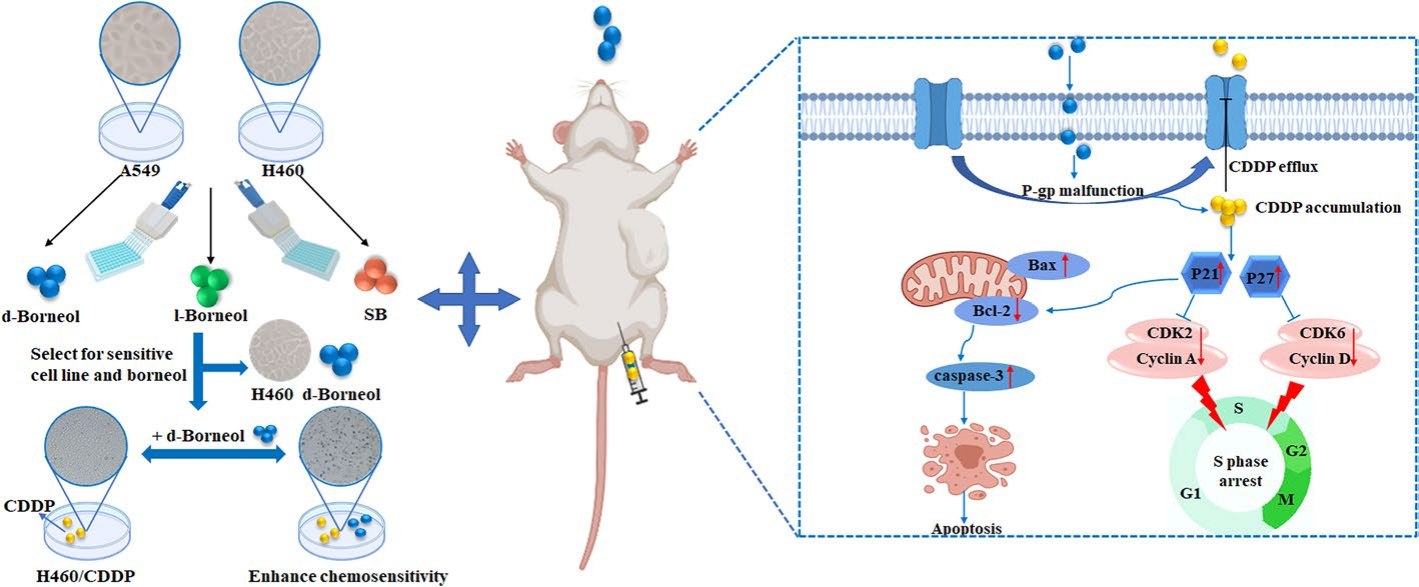
Schematic diagram of antitumor effect in vivo and in vitro and the proposed signaling pathway induced by combination treatment with d-borneol and CDDP. d-Borneol enhanced the sensitivity of CDDP by increasing CDDP cellular accumulation via inhibiting P-gp function. On the one hand, d-borneol enhanced p21 and p27 and suppressed two complexes, i.e., CDK2–cyclin A and CDK6–cyclin D, which blocked the cell cycle in S phase. On the other hand, d-borneol enhanced the sensitivity of CDDP to H460/CDDP cells by promoting the mitochondrially mediated intrinsic apoptosis via activating pro-apoptotic Bax, downregulating anti-apoptotic Bcl-2, and finally promoting activation of caspase-3, which leads to apoptosis. “←” indicates activation, and “⟝” indicates inhibition

We showed for the first time that H460 cells exhibit higher sensitivity to d-borneol than A549 cells. Further, combined application of d-borneol and CDDP enhanced cell sensitivity through activating intrinsic apoptosis via p21-mediated Bax/Bcl-2/caspase-3 signaling and arresting the cell cycle at S phase via p27/p21-mediated cyclinA2/D3-CDK2/6. These findings support the reliability of RNA-seq results and our initial hypothesis. Our study has also found that d-borneol reduced the expression of P-gp and inhibited drug efflux, thereby increasing sensitivity to CDDP. In vivo, d-borneol combined with CDDP inhibited NSCLC tumor growth significantly and ameliorated CDDP toxicity (Fig. 8), which reflects the advantages of increasing efficacy and reducing toxicity by combining natural products and chemotherapeutic drugs. Biological mechanisms of tumor occurrence and development are complicated, and much is still unknown. Therefore, we will focus on the key biological mechanisms in vivo to provide experimental evidence that d-borneol is potential chemosensitizer for the treatment of lung cancer.

## Supplementary Information

**Additional file 1:** The STR profiling of A549 cell.

**Additional file 2:** The STR profiling of H460 cell.

## Data Availability

The original contributions presented in the study are included in the article; further inquiries can be directed to the corresponding author.

## Abbreviations

ANOVA: One-way analysis of variance
BBB: Blood–brain barrier
CDDP: Cisplatin
CDK: Cyclin-dependent kinases
CKI: Cyclin-dependent kinase inhibitor
MDR: Multidrug resistance
NSCLC: Non-small cell lung cancer
P-gp: P-glycoprotein
Rho 123: Rhodamine 123
RNA-seq: RNA sequencing
SB: Synthetic borneol
TCM: Traditional Chinese medicine
