# Correction: d-Borneol enhances cisplatin sensitivity via p21/p27-mediated S-phase arrest and cell apoptosis in non-small cell lung cancer cells and a murine xenograft model

**DOI:** 10.1186/s11658-022-00373-1

**Published:** 2022-08-18

**Authors:** Jinxiu Li, Jianmei Yuan, Yong Li, Jian Wang, Daoyin Gong, Qian Xie, Rong Ma, Jiajun Wang, Mihong Ren, Danni Lu, Zhuo Xu

**Affiliations:** 1 State Key Laboratory of Southwestern Chinese Medicine Resources, College of Pharmacy, Chengdu University of Traditional Chinese Medicine, Chengdu, China; 2 Department of Pathology, Hospital of Chengdu University of Traditional Chinese Medicine, Chengdu, China

## Correction: Cellular & Molecular Biology Letters (2022) 27:61 https://doi.org/10.1186/s11658-022-00362-4

Following publication of the original article [1], the authors informed us that would like to replace the Figure 1L. The correct Fig. 1 is given below:

**Fig. 1:**
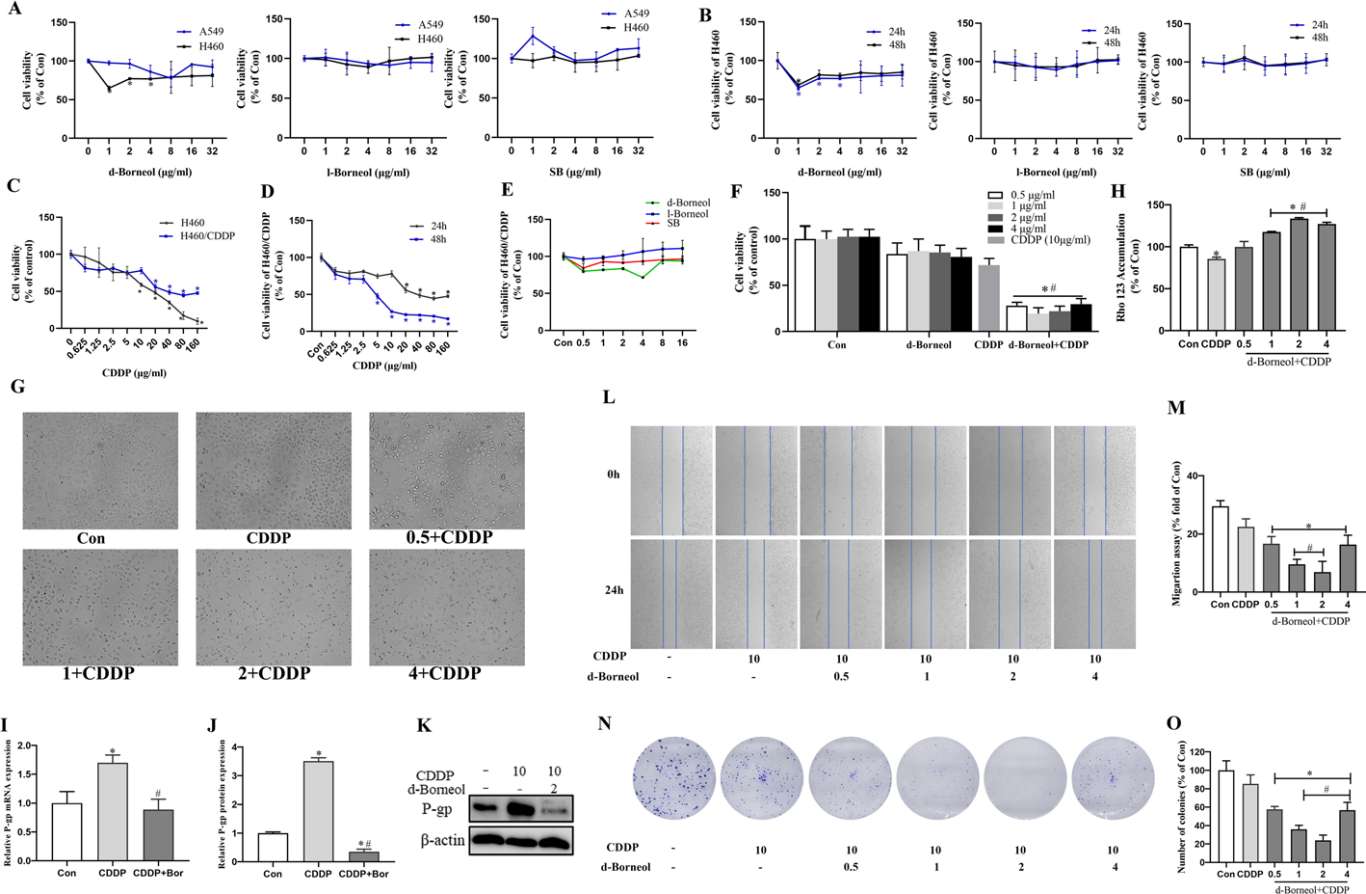
Cell viability of different NSCLC cells treated with three borneol forms and cotreatment with d-borneol and CDDP, and effect on cell proliferation, P-gp function, and cell migration in H460/CDDP cells. **A** A549 and H460 cells cultured with different concentrations of d-borneol, l-borneol, and SB for 24 h; **B** H460 was treated with d-borneol, l-borneol, and SB for 24 h and 48 h, and cell viability was determined by MTT; **C** cell viability of H460 and H460/CDDP cells treated with diferent concentrations of CDDP for 24 h; **D** cell viability of CDDP on H460/CDDP cells for 24 h and 48 h; **E** cytotoxicity of the three borneol forms on H460/CDDP cells; F, G cell viability (**F**) and cell morphology (**G**) of H460/CDDP cells treated with CDDP (10 μg/ml), or d-borneol (0.5, 1, 2, and 4 μg/ml) + CDDP (10 μg/ml) for 24 h; **H** determination of P-gp function after treatment with CDDP, CDDP plus d-borneol with different concentrations for 24 h; **I**, **K** mRNA expression (**I**) and protein expression (**J**, **K**) of P-gp; **L**,** M** cell migration of H460/CDDP cells; **N** representative photographs showing colony formation after CDDP cotreatment with d-borneol in H460/CDDP cells; **O** calculation of statistical differences. All data are presented as mean ± SD (n = 3, *P < 0.05, compared with Con; ^#^*P* < 0.05, compared with CDDP group)

Also, an error was identified in the **Results** section.

The updated phrase is given below and the changes have been highlighted in **bold typeface**.

## Results

### d-Borneol combined with CDDP suppresses NSCLC tumor growth in vivo

We noted that treatment with d-borneol alone caused no notable body weight change, but the body weight of animals treated with CDDP was significantly lower than that of those in the Con group. After 14 days of treatment with the combination of d-borneol and CDDP, the CDDP + Bor group increased body weight significantly compared with the CDDP group, indicating that d-borneol **weaken** the toxicity of cisplatin in vivo (Fig. 6E) 0.2111.

The original article has been corrected.
