# Supplementary material for: d-Borneol enhances cisplatin sensitivity via p21/p27-mediated S-phase arrest and cell apoptosis in non-small cell lung cancer cells and a murine xenograft model
Source: Cell Mol Biol Lett. 2022 Jul 26;27:61. doi: 10.1186/s11658-022-00362-4 (PMC9327246; doi:10.1186/s11658-022-00362-4)

Cell Line Authentication – STR Profiling

Sample from: FuHeng Cell Center, Shanghai, China

Testing Method: STR Genotyping

Report Time:May 20, 2019

Cell Line Authentication – STR Profiling Report

Sample code

Table 1. Sample Code

Customer's code	Company Code
A549	20190510-02

Sample Number:1

Sample Type: Cell line

Testing Type: STR

Sample From: FuHeng Cell Center, Shanghai, China

Testing Method:

DNA was extracted by a commercial kit from CORNING (AP-EMN-BL-GDNA-250G). The twenty STRs including Amelogenin locus were amplified by six multiplex PCR and separated on ABI 3730XL Genetic Analyzer. The signals were then analyzed by the software GeneMapper.

Data Interpretation:

Cell lines were authenticated using Short Tandem Repeat (STR) analysis as described in 2012 in ANSI Standard (ASN-0002) by the ATCC Standards Development Organization (SDO) and inCapes-Davis et al., Match criteria for human cell line authentication: Where do we draw the line?

Int J Cancer.2013;132(11):2510-9.

Test Results:

1. Result

Table 2. Matching information on the cell lines

Sample Code	Multi-allele	Cell line matched	Cell Bank	Percentage
20190510-02	NO	A549	DMSZ	9/9

Multi-allele means some STR contain more than two loci.

2. Sample Description

20190510-02 The DNA of the cell lines found to basic match the type of cell lines in a cell lineretrieval, DMSZ database shows that cells called A549 corresponding to the cell number **ACC-107**. No multiple alleles were found in this cell line.

3. Genotyping Result

STR and Amelogenin Genotyping Results of Cell line 20190510-02						
Loci	**Sample**: A549			**Cell Bank information**: A549		
	Allele1	Allele2	Allele3	Allele1	Allele2	Allele3
D5S818	11	11		11	11	
D13S317	11	11		11	11	
D7S820	8	11		8	11	
D16S539	11	12		11	12	
VWA	14	14		14	14	
TH01	8	9.3		8	9.3	
AMEL	X	Y		X	Y	
TPOX	8	11		8	11	
CSF1PO	10	12		10	12	

D12S391	18	18				
FGA	23	23				
D2S1338	24	24				
D21S11	29	29				
D18S51	14	17				
D8S1179	13	14				
D3S1358	16	16				
D6S1043	10	11	13			
PENTAE	7	11				
D19S433	13	13				
PENTAD	7	9				

Others

1. Genotyping Strategy and Site DistributionAttached Table. Experimental Strategy and Sites

	**Strategy 1**	**Strategy 2**	**Strategy 3**	**Strategy 4**
1	TH01	TPOX	D3S1358	AMEL
2	D12S391	VWA	D13S317	D5S818
3	D7S820	D8S1179	D6S1043	D2S1338
4	CSF1PO	PENTAD	D16S539	D21S11
5	FGA		D19S433	D18S51

6	PENTAE			

The allele match algorithm compares the 8 core loci plus amelogenin only, even though alleles from all loci will be reported when available.

2. STR database comparison

DSMZ tools was used to carry on the cell line comparison, which contains 2455 cell lines STR data from ATCC, DSMZ, JCRB ,ECACC and RIKEN databases. If the cell is not included in the above cell library, users need to compared with other databases.

Sample File	Sample Name	Panel	SQO	OS	SQ
47_G06_CellLineAuthehntication-1-0516.fsa	A549	21Plex_STR_Panel_v0.4			

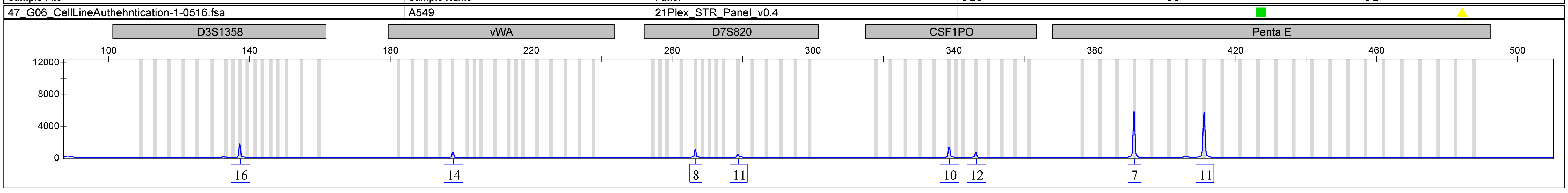

Sample File	Sample Name	Panel	SQO	OS	SQ
47_G06_CellLineAuthehntication-1-0516.fsa	A549	21Plex_STR_Panel_v0.4			

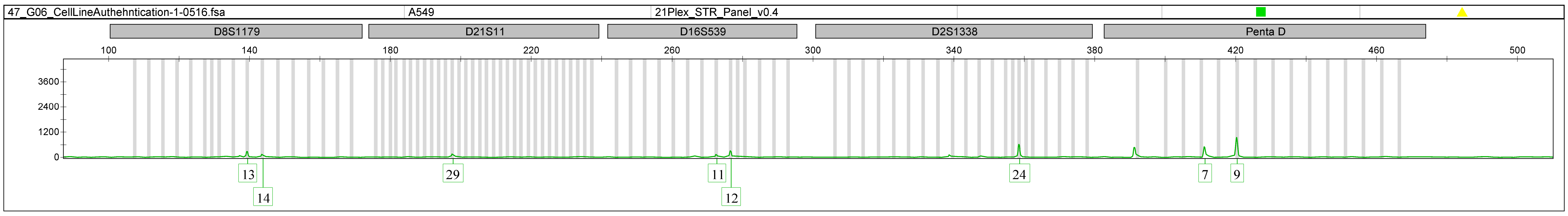

Sample File	Sample Name	Panel	SQO	OS	SQ
47_G06_CellLineAuthehntication-1-0516.fsa	A549	21Plex_STR_Panel_v0.4			

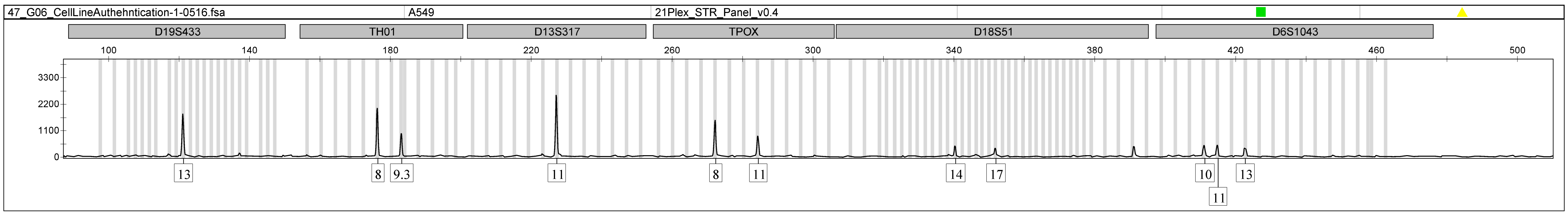

Sample File	Sample Name	Panel	SQO	OS	SQ
47_G06_CellLineAuthehntication-1-0516.fsa	A549	21Plex_STR_Panel_v0.4			

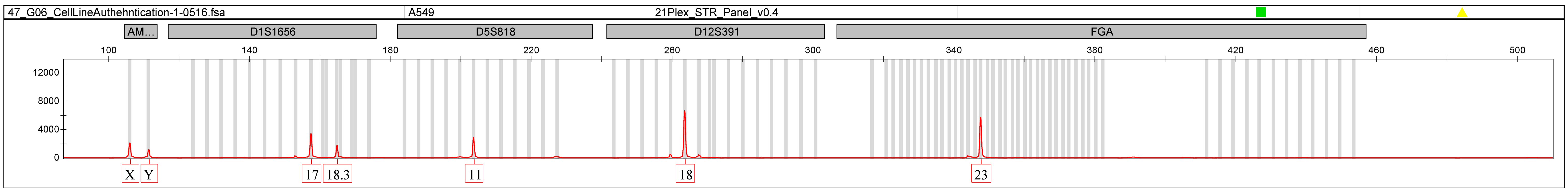

Supplement: Supplementary file 1 — Additional file 1: The STR profiling of A549 cell. [file 11658_2022_362_MOESM1_ESM.pdf]
