# Supplementary material for: d-Borneol enhances cisplatin sensitivity via p21/p27-mediated S-phase arrest and cell apoptosis in non-small cell lung cancer cells and a murine xenograft model
Source: Cell Mol Biol Lett. 2022 Jul 26;27:61. doi: 10.1186/s11658-022-00362-4 (PMC9327246; doi:10.1186/s11658-022-00362-4)

Cell Line Authentication – STR Profiling

Sample from: FuHeng Cell Center, Shanghai, China
Testing Method: STR Genotyping
Report Time:May 13, 2022

Cell Line Authentication - STR Profiling Report

Sample code

Table 1. Sample Code

Customer's code	Company Code
GLCI-1	20220511-01

Sample Number:1

Sample Type: Cell line

Testing Type: STR

Sample From: FuHeng Cell Center, Shanghai, China

Testing Method:

DNA was extracted by a commercial kit from CORNING (AP-EMN-BL-GDNA-250G). The twenty STRs including Amelogenin locus were amplified by six multiplex PCR and separated on ABI 3730XL Genetic Analyzer. The signals were then analyzed by the software GeneMapper.

Data Interpretation:

Cell lines were authenticated using Short Tandem Repeat (STR) analysis as described in 2012 in ANSI Standard (ASN-0002) by the ATCC Standards Development Organization (SDO) and inCapes-Davis et al., Match criteria for human cell line authentication: Where do we draw the line?

Int J Cancer.2013;132(11):2510-9.

Test Results:

1. Result

Table 2. Matching information on the cell lines

Sample Code	Multi-allele	Cell line matched	Cell Bank	Percentage
20220511-01	NO	NCI-H460	DSMZ	1.0

Multi-allele means some STR contain more than two loci.

2. Sample Description

20220511-01: The DNA typing of the cell line found a perfectly matched cell line in the cell line search. The DSMZ database showed that the cell name was NCI-H460 and the cell number corresponded to 737. No multiple alleles were found in this cell line.

3. Genotyping Result

STR and Amelogenin Genotyping Results of Cell line 20220511-01						
Loci	**Sample**: GLCI-1			**Cell Bank information**: NCI-H460		
	Allele1	Allele2	Allele3	Allele1	Allele2	Allele3
D5S818	9	10		9	10	
D13S317	13	13		13	13	
D7S820	9	12		9	12	
D16S539	9	9		9	9	
VWA	17	17		17	17	
TH01	9.3	9.3		9.3	9.3	
AMEL	X	Y		X	Y	
TPOX	8	8		8	8	
CSF1PO	11	12		11	12	
D12S391	21	21				
FGA	21	23				

D2S1338	17	25				
D21S11	30	30				
D18S51	13	15				
D8S1179	12	12				
D3S1358	15	18				
D6S1043	11	14				
PENTAE	5	5				
D19S433	14	14				
PENTAD	11	13				
D1S1656	11	15				

Others

1. Genotyping Strategy and Site DistributionAttached Table. Experimental Strategy and Sites

	**Strategy 1**	**Strategy 2**	**Strategy 3**	**Strategy 4**
1	TH01	TPOX	D3S1358	AMEL
2	D12S391	VWA	D13S317	D5S818
3	D7S820	D8S1179	D6S1043	D2S1338
4	CSF1PO	PENTAD	D16S539	D21S11
5	FGA		D19S433	D18S51
6	PENTAE			

The allele match algorithm compares the 8 core loci plus amelogenin only, even though alleles from all loci will be reported when available.

2. STR database comparison

DSMZ tools was used to carry on the cell line comparison, which contains 2455 cell lines STR data from ATCC, DSMZ, JCRB ,ECACC and RIKEN databases. If the cell is not included in the above cell library, users need to compared with other databases.

Sample File	Sample Name	Panel	OS	SQ
53_E07_CellLineAuthentication-2--0512.fsa	GLCI-1	21Plex_STR_Panel_v1.1		

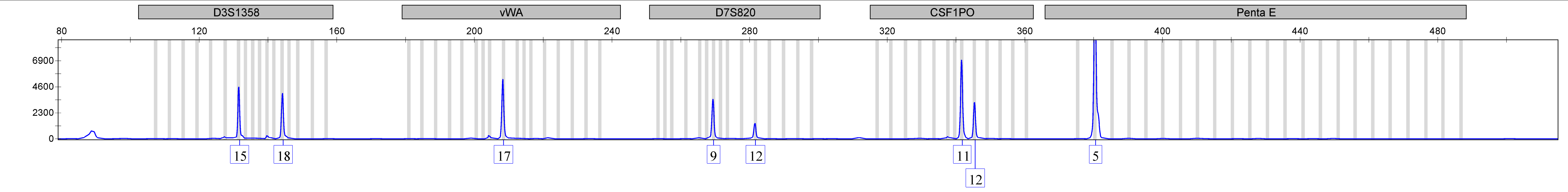

53_E07_CellLineAuthentication-2--0512.fsa	GLCI-1	21Plex_STR_Panel_v1.1		

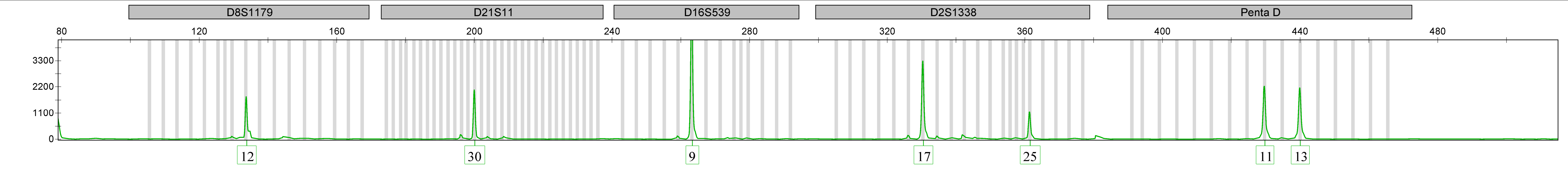

53_E07_CellLineAuthentication-2--0512.fsa	GLCI-1	21Plex_STR_Panel_v1.1		

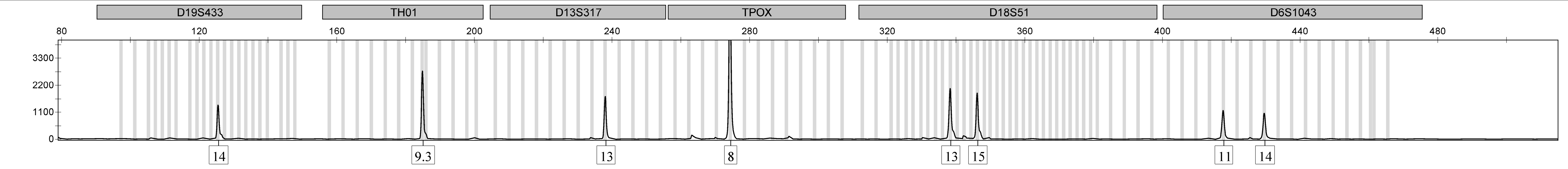

53_E07_CellLineAuthentication-2--0512.fsa	GLCI-1	21Plex_STR_Panel_v1.1		

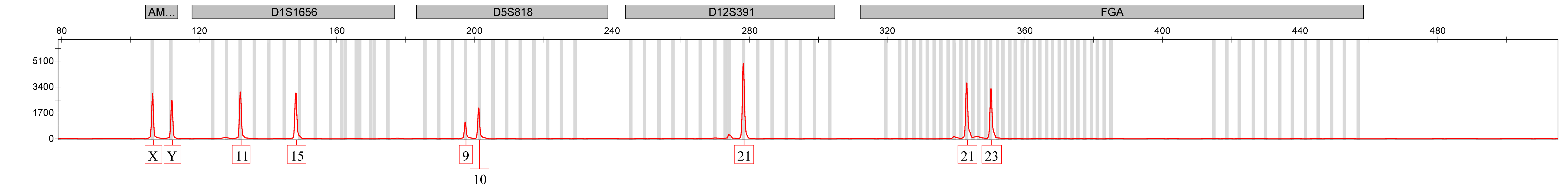

Supplement: Supplementary file 2 — Additional file 2: The STR profiling of H460 cell. [file 11658_2022_362_MOESM2_ESM.pdf]
